# AI-Based Sepsis Prediction in Hospitalized Adults: Systematic Review, Subgroup Meta-Analysis, and Contextual Analysis of Clinical Burden

**DOI:** 10.2196/95665

**Published:** 2026-09-17

**Authors:** Gyeong Min Lee, Joo-Yun Won, Eun Young Cho, Ji-Hyun Kim, Kwang Joon Kim, Yu Seung Lee, Hyun Jun Lee, Jae Hyun Kim

**Affiliations:** 1 Research Institute for Healthcare Policy, Dankook University, Cheonan, Chungcheongnam-do, Republic of Korea; 2 AITRICS Corp, Seoul, Republic of Korea; 3 Department of Health Administration, College of Public Health Sciences, Dankook University, Dongnam-gu, 119, Dandae-ro, Cheonan-si, Chungcheongnam-do, 31116, Republic of Korea, 82 10-8438-8353, 82 41-559-7934

**Keywords:** sepsis, machine learning, deep learning, AI, prediction model, systematic review, meta-analysis, clinical burden, claims data

## Abstract

**Background:**

Machine learning (ML) and deep learning (DL) models have been developed for earlier recognition in hospitalized patients, but reported performance varies across datasets, prediction windows, care settings, and validation designs. Interpretation of a single pooled discrimination estimate is therefore uncertain, particularly because public datasets are often reused, and most evidence is retrospective.

**Objective:**

This study aimed to synthesize the performance of ML- and DL-based sepsis prediction models in hospitalized adults, emphasizing prediction windows and validation maturity, and to separately describe sepsis-related health care burden using Korean national inpatient claims data.

**Methods:**

We conducted a systematic review and meta-analysis of ML and DL models for sepsis prediction in hospitalized adults. The protocol was registered in PROSPERO. Random-effects meta-analysis used the Hartung-Knapp-Sidik-Jonkman approach, with 95% prediction intervals where sufficient studies were available. Interpretation focused on prediction-window subgroups and validation-maturity tiers rather than a single pooled area under the receiver operating characteristic curve (AUROC). Potential nonindependence from repeated use of Medical Information Mart for Intensive Care (MIMIC) and PhysioNet cohorts was examined through dataset-overlap assessment and sensitivity analysis. Separately, Korean Health Insurance Review and Assessment Service National Inpatient Sample data were used to describe length of stay, medical costs, and surgery counts by sepsis-related episode timing; this analysis did not validate an AI model.

**Results:**

In total, 34 studies were included, most of which were retrospective model-development or validation studies. Several reused MIMIC- or PhysioNet-derived cohorts, so the 34 reports did not represent 34 fully independent patient populations. The pooled AUROC was 0.913 (95% CI 0.887‐0.933), with a 95% prediction interval of 0.660‐0.983. In exploratory subgroup analyses, pooled AUROCs were 0.894 (95% CI 0.829‐0.936) for models predicting sepsis within 4 hours, 0.926 (95% CI 0.897‐0.948) for models predicting more than 4 hours before onset, and 0.858 (95% CI 0.581‐0.964) for unclear or unreported prediction windows. Overlapping prediction intervals indicated substantial uncertainty and did not establish superiority of any prediction horizon. Prospective, randomized, and implementation studies were interpreted separately. In the Korean claims analysis, sepsis-related episode groups showed longer observed hospital stays and higher unadjusted medical costs than general inpatient episodes.

**Conclusions:**

Reported ML and DL sepsis prediction models frequently demonstrated good discrimination within individual study settings, but performance in new clinical populations remains uncertain because of extreme heterogeneity, overlapping public data, inconsistent reporting, and limited prospective evaluation. Prediction-window and validation-maturity analyses were more clinically informative than a single pooled AUROC, although exploratory. The Korean claims analysis provided separate contextual evidence of disease burden and should not be interpreted as AI model validation.

## Introduction

Sepsis is a life-threatening organ dysfunction caused by a dysregulated host response to infection. Importantly, sepsis-related mortality can occur without septic shock, reflecting complex and heterogeneous pathophysiology [1,2]. Due to its heterogeneous presentation—shaped by factors such as age, comorbidities, and infection source—early diagnosis remains challenging [3]. The high morbidity, rapid progression, and lack of early symptoms contribute to delays in recognition, making early detection a critical global health priority [4].

In 2020, about 48.9 million cases of sepsis and 11 million related deaths were estimated globally, accounting for 20% of all deaths [5]. The incidence continues to rise due to aging populations, increasing prevalence of chronic illnesses, immunosuppressive therapies, and antimicrobial resistance [6,7]. Mortality rates range from 10% to 20% in sepsis, 20% to 40% in severe sepsis, and 40% to 80% in septic shock [8,9].

The financial burden is also immense. Sepsis represents the largest portion of disease-related hospital costs in the United States [10]. Beyond direct hospitalization and treatment expenses, indirect costs—such as reduced productivity, long-term complications, and quality-of-life impairments—add further strain to both survivors and health care systems [11,12]. Early detection and treatment significantly reduce these burdens and improve outcomes [13-15], with timely recognition being one of the most effective strategies to mitigate both clinical and economic consequences [10,11].

Traditional clinical tools, such as Sequential Organ Failure Assessment (SOFA) [16-18], and regression analysis [19,20] are designed to quantify organ dysfunction rather than to predict future sepsis onset. However, these approaches face limitations. SOFA requires multiple variables, making it less suitable for rapid decision-making [21], and biomarker-based models often require evaluating multiple indicators rather than a single reliable predictor [18]. Additionally, regression models struggle to capture the complex, nonlinear interactions inherent in sepsis [20].

There has been increasing research interest in applying machine learning (ML) approaches to improve the accuracy and timeliness of sepsis prediction [22]. ML, a subset of AI, uses algorithms and statistical models to identify patterns in complex datasets and make predictions [23,24]. In sepsis prediction, ML can integrate diverse data such as vital signs, laboratory results, and clinical notes to detect early warning signs that traditional tools may overlook [20,22].

In addition to synthesizing published model-performance studies, this study included a complementary descriptive burden analysis using Korean national inpatient claims data. This claims-based component was not designed to externally validate an AI-based sepsis prediction model [22,25]. Instead, it was used to describe health care use and clinical burden according to sepsis-related episode timing, including length of stay, total medical cost, and surgery count.

The primary objective of this study was to synthesize reported performance of ML and deep learning (DL)–based sepsis prediction models in hospitalized adults, with particular emphasis on clinically relevant prediction windows, validation maturity, and the extent to which performance estimates were derived from independent patient cohorts. A secondary and analytically separate objective was to describe the clinical and economic burden associated with sepsis-related episode timing using Korean national inpatient claims data. By separating retrospective model-performance evidence from prospective implementation evidence and contextual burden information, we aimed to provide a more clinically interpretable assessment of the current sepsis AI evidence base.

## Methods

### Study Design

This study conducted a systematic review and meta-analysis to evaluate the predictive accuracy of ML and DL models for predicting the onset of sepsis in hospitalized patients across all care settings, including general wards, intensive care units (ICUs), and emergency departments (EDs). The systematic review protocol was prospectively registered with PROSPERO (registration: CRD420251005274).

### Search Strategy

A comprehensive literature search was conducted to identify studies evaluating AI, ML, or DL models for sepsis prediction in hospitalized adult patients. The initial search was performed in Embase, MEDLINE, and the Cochrane Library up to March 12, 2025. Search terms combined concepts related to AI and predictive modeling with sepsis-related terms. The core search strategy included terms such as “artificial intelligence,” “machine learning,” “deep learning,” “sepsis,” “septic shock,” “sepsis prediction,” and “sepsis onset prediction.” The detailed database-specific search queries are provided in Table S1 in Multimedia Appendix 1.

To better capture deployment-relevant and landmark sepsis AI studies, we conducted an additional targeted search using model names, implementation terms, and landmark-study terms. These included “early warning,” “clinical deterioration,” “decision support,” “real-time alert,” “TREWS,” “Targeted Real-Time Early Warning System,” “Epic Sepsis Model,” “NAVOY,” “COMPOSER,” “Sepsis ImmunoScore,” “InSight,” and related terms. This targeted search was intended to identify prospective implementation studies, randomized or quasi-experimental evaluations, external validation studies, and regulatory or commercial-model evaluations that may not have been retrieved by generic AI and ML search terms alone.

All retrieved records were exported to EndNote (Clarivate) and screened after duplicate removal. The search and screening process followed PRISMA (Preferred Reporting Items for Systematic Reviews and Meta-Analyses) 2020 principles, and the completed PRISMA 2020 checklist is provided in Checklist 1. An AI-specific PRISMA reporting addendum is provided in Table S4 in Multimedia Appendix 1. Records were first screened by title and abstract, followed by full-text assessment for potentially eligible studies. Studies identified through targeted searches were assessed using the same eligibility criteria as the main search. If a deployment or validation study did not meet the criteria for quantitative pooling because of differences in study objective, outcome definition, intervention design, or reported performance metrics, it was retained for narrative synthesis and interpretation of clinical implementation evidence where relevant. Additional recent studies identified during paper revision were used only for contextual discussion and were not included in the quantitative synthesis.

### Study Selection

After duplicate removal, 2 reviewers (YSL and GML) independently screened titles and abstracts to identify potentially eligible studies. Disagreements at the title-and-abstract stage were resolved through discussion. Records judged as potentially relevant by either reviewer were retained for further assessment to minimize the risk of excluding clinically important studies at the screening stage.

Full-text papers were then reviewed independently by 2 reviewers (YSL and GML) using the predefined inclusion and exclusion criteria. Disagreements during full-text assessment were resolved by consensus, and unresolved cases were adjudicated by a senior reviewer. When multiple papers reported overlapping datasets or different versions of the same prediction model, we retained the paper with the most complete information on model performance, validation design, or clinical implementation. For studies that were important for clinical interpretation but not suitable for quantitative synthesis, such as randomized evaluations, prospective implementation studies, quasi-experimental deployment studies, regulatory validation studies, or external validation studies with different outcomes, we summarized them narratively rather than combining them directly with retrospective model-development studies.

We additionally reviewed dataset provenance to identify potentially overlapping cohorts. The included evidence contained 1 study using Medical Information Mart for Intensive Care (MIMIC)-II, 9 studies including MIMIC-III, 4 studies including MIMIC-IV, and 3 studies identified as using the PhysioNet 2019 Challenge dataset. Because studies using the same public dataset family were not fully independent at the patient level, dataset reuse was documented and considered in sensitivity analyses and interpretation. Studies evaluating distinct model architectures on shared datasets were retained in the descriptive evidence table, but the number of included reports was not interpreted as the number of independent clinical cohorts.

Reasons for full-text exclusion were recorded and categorized as wrong population, nonsepsis outcome, nonpredictive study design, method not based on AI or ML, pediatric or neonatal population, review or commentary, conference abstract only, duplicate or overlapping dataset, insufficient model-performance information, or not suitable for quantitative model-performance synthesis.

### Inclusion and Exclusion Criteria

Eligible studies were original, peer-reviewed papers describing AI-based predictive models for sepsis onset in hospitalized adult patients. Included studies clearly defined sepsis and the predictive modeling techniques used. Excluded were reviews, meta-analyses, nonhuman studies, and studies without clear predictive outcome definitions. Studies exclusively focused on pediatric, neonatal, or outpatient populations were also excluded.

### Data Extraction

Data were extracted independently by 2 reviewers (YSL and GML) using a predefined extraction form. Extracted information included study characteristics, country, publication year, study design, care setting, data source, sample size, sepsis definition, sepsis prevalence, baseline SOFA operationalization where reported, prediction window, model type, input variables, missing-data handling, imputation strategy, validation approach, and performance metrics including area under the receiver operating characteristic curve (AUROC), sensitivity, specificity, accuracy, positive predictive value, and negative predictive value where available.

After independent extraction, the 2 reviewers (YSL and GML) compared the extracted data. Discrepancies were resolved through discussion and rechecking of the original papers. If disagreement persisted, a senior reviewer made the final decision. When information was unclear or not reported in the original study, it was recorded as “not reported” rather than inferred. For quantitative synthesis, we extracted the primary model performance estimate reported by each study. If multiple models were presented, we used the best-performing clinically relevant model or the model identified by the study authors as the primary model.

For each study, we also recorded dataset provenance and assigned the study to a dataset family, including MIMIC-II, MIMIC-III, MIMIC-IV, PhysioNet 2019 Challenge, other public datasets, or institution-specific datasets. When a study used multiple public datasets, all relevant dataset families were recorded. This information was used to identify potential cohort overlap and to conduct sensitivity analyses based on dataset independence.

For dataset-family sensitivity analyses, we retained one report per overlapping public dataset family. The retained report was selected hierarchically according to external validation status, multicenter design, completeness of uncertainty reporting, and sample size.

### Risk-of-Bias and Applicability Assessment

Risks of bias and applicability were assessed using a Prediction Model Risk of Bias Assessment Tool (PROBAST)+AI-oriented framework for AI- and ML-based prediction model studies. The assessment focused on domains relevant to prediction-model validity and clinical applicability, including participants and data sources, predictors, outcome definition, model analysis, missing-data handling, validation design, calibration or threshold reporting, transparency of preprocessing and feature handling, and applicability to the intended inpatient sepsis prediction context.

Two reviewers (YSL and GML) independently assessed each included study. Disagreements were resolved through discussion, and unresolved conflicts were adjudicated by a senior reviewer. Studies were not excluded solely on the basis of risk-of-bias or applicability concerns. Instead, the assessment was used to guide interpretation of the evidence, particularly because many included studies were retrospective, varied in sepsis definitions and care settings, and incompletely reported calibration, external validation, and missing-data handling.

In response to concerns regarding clinical and methodological heterogeneity, we additionally extracted sepsis definition, baseline SOFA operationalization where reported, care setting, prediction window, sepsis prevalence, missing-data reporting, and imputation strategy. Care setting was classified as ICU, ED, general ward, mixed inpatient setting, or not reported. Sepsis definition was classified as Sepsis-2, Sepsis-3, *International Classification of Diseases* (ICD) code–based, institution-specific, or unclear or not reported. Baseline SOFA handling was extracted when studies used Sepsis-3 criteria or described SOFA-based outcome labeling. Missing-data handling was categorized according to whether the study reported complete-case analysis, single imputation, multiple imputation, forward filling, model-based imputation, other approaches, or did not report the strategy.

### Statistical Analysis and Meta-Analysis

The primary quantitative outcome was the AUROC because it was the most consistently reported discrimination measure across the included studies. Study-specific AUROC estimates and 95% CIs were extracted where available. SEs were derived from reported CIs using the normal approximation. When sufficient uncertainty information was unavailable, this was recorded and considered in the interpretation of the synthesis.

AUROC values were logit-transformed before quantitative synthesis to ensure that pooled estimates and prediction intervals remained within the permissible range of 0 to 1. Results were subsequently back-transformed to the original AUROC scale for presentation. Because substantial clinical and methodological heterogeneity was anticipated, random-effects meta-analysis was performed using restricted maximum likelihood estimation [26], with the Hartung-Knapp-Sidik-Jonkman (HKSJ) adjustment for statistical inference [27]. This approach accounts for uncertainty in the estimated between-study variance and generally provides more conservative inference than the conventional normal-approximation random-effects method.

Heterogeneity was assessed using τ^2^, *I*^2^, and visual inspection of forest plots. Because *I*^2^ describes the proportion of variability attributable to between-study heterogeneity but does not indicate the expected range of true effects across different clinical settings, 95% prediction intervals were calculated for the overall analysis and subgroup analyses containing a sufficient number of studies. Prediction intervals were interpreted as the approximate range within which the underlying discrimination of a comparable model in a new population or clinical setting might be expected to fall.

The overall pooled AUROC was treated as a secondary descriptive summary. Primary interpretation focused on clinically relevant subgroups, particularly prediction window and validation maturity. Prediction-window categories were defined as models predicting sepsis within 4 hours before onset or clinical confirmation, models predicting sepsis more than 4 hours before onset, and studies with an unclear or unreported prediction window. Because the 4-hour threshold was not prespecified in the PROSPERO protocol, this subgroup analysis was considered exploratory and post hoc.

Evidence was also classified according to validation maturity: retrospective model development or internal validation, retrospective external validation, prospective observational or real-time implementation, quasi-experimental deployment, and randomized clinical evaluation. Studies evaluating clinical implementation or patient outcomes using substantially different designs and outcome measures were summarized narratively rather than pooled with retrospective AUROC-based model-performance studies.

To examine potential nonindependence caused by repeated use of public cohorts, a dataset-family sensitivity analysis was conducted. Studies using the same public dataset family, including MIMIC-II, MIMIC-III, MIMIC-IV, and the PhysioNet 2019 Challenge dataset, were considered potentially nonindependent. In the sensitivity analysis, each overlapping dataset family was represented by one prespecified study. The representative study was selected hierarchically according to external-validation status, multicenter design, completeness of AUROC uncertainty reporting, and sample size. Studies based on institution-specific or otherwise independent datasets were retained. The sensitivity-analysis results were compared with those of the primary report-level analysis to assess whether repeated use of public datasets materially affected the pooled estimate, CI, or prediction interval.

A bivariate or hierarchical summary receiver operating characteristic model was considered but was not used as the primary analysis because threshold-specific sensitivity, specificity, corresponding CIs, and 2×2 table information were not consistently reported. Accordingly, the AUROC synthesis was interpreted as a summary of discrimination rather than diagnostic accuracy at a common clinical decision threshold.

To assess potential publication bias or small-study effects, Deeks funnel plot asymmetry testing was conducted among studies with sufficient threshold-specific information to estimate diagnostic odds ratios. Because only a subset of the included studies could be evaluated, this analysis was considered exploratory. The Deeks asymmetry test was 2-sided, with *P*<.05 indicating statistically detectable asymmetry. All analyses were performed using R (version 4.2.0; R Foundation for Statistical Computing) with the *metafor* package (version 4.6‐0) for random-effects meta-analysis, HKSJ inference, and prediction intervals, and the *meta* package (version 7.0‐0) for supplementary forest plots and sensitivity analyses.

### Exploratory Descriptive Analysis of Clinical Burden Using the National Inpatient Sample

In addition to the meta-analysis of published studies, we conducted a descriptive analysis of clinical burden using the National Inpatient Sample from the Health Insurance Review and Assessment Service of Korea (HIRA-NIS 2020, S20241108001). This analysis was not designed to externally validate any AI-based sepsis prediction model. Instead, it was conducted to provide contextual evidence on health care use and clinical burden according to sepsis-related episode timing.

The HIRA-NIS dataset was preprocessed through a structured pipeline that included identification of eligible inpatient episodes, application of sepsis-related diagnosis codes, episode construction, duplicate claim removal, and time-window matching. Sepsis was identified using ICD primary diagnosis codes A40.0-A40.3, A40.8-A40.9, A41.0-A41.5, and A41.8-A41.9. Based on the episode-construction process, patients were classified into analytic groups according to sepsis-related timing, including general inpatient episodes, sepsis episodes, prior sepsis followed by a subsequent inpatient episode, and inpatient episodes followed by subsequent sepsis.

**Figure 1.**
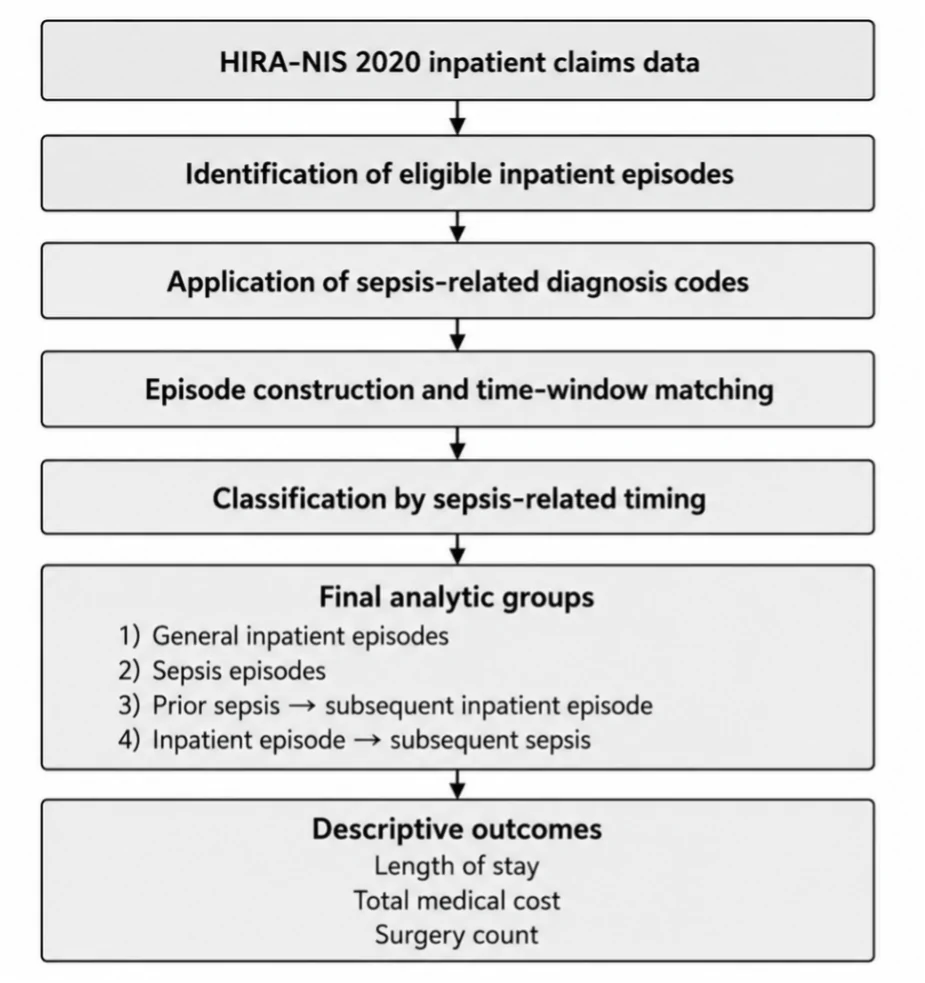
Simplified flowchart of HIRA-NIS data processing and episode classification for descriptive sepsis burden analysis. HIRA-NIS: National Inpatient Sample from the Health Insurance Review and Assessment Service of Korea.

Figure 1 presents a simplified overview of the HIRA-NIS data-processing and episode-classification procedure. To improve readability, the revised figure focuses on the main analytic steps rather than detailed counts at each processing stage. Detailed preprocessing steps and numerical information on exclusions, episode construction, and sepsis-related case identification are described in the text rather than embedded within the figure.

### Ethical Considerations

The systematic review used data from previously published studies and did not require separate institutional review board approval. The Korean claims-based descriptive analysis was approved by the institutional review board of Dankook University (IRB DKU 2024-11-043-001). The requirement for informed consent was waived because the analysis used deidentified secondary claims data obtained from the Health Insurance Review and Assessment Service of Korea. No directly identifiable individual-level information was accessed.

## Results

### Study Selection

**Figure 2.**
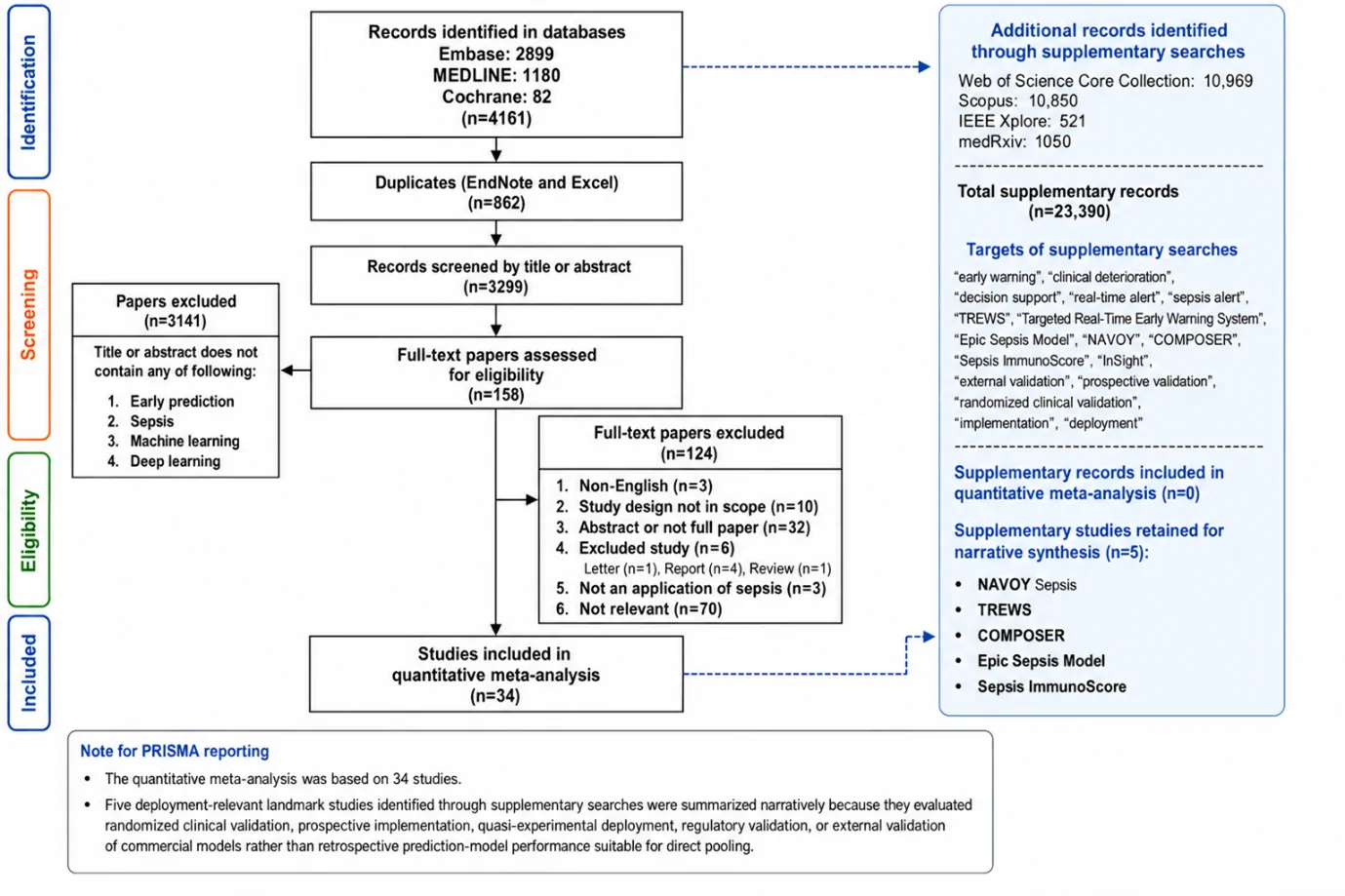
Literature screening flowchart. Supplementary search records were used to identify landmark implementation and validation studies and were not merged into the primary PRISMA screening denominator. Supplementary studies retained for narrative synthesis included NAVOY Sepsis, TREWS, COMPOSER, the Epic Sepsis Model, and Sepsis ImmunoScore. COMPOSER: Conformal Multidimensional Prediction of Sepsis Risk; PRISMA: Preferred Reporting Items for Systematic Reviews and Meta-Analyses; TREWS: Targeted Real-Time Early Warning System.

The initial database search identified 4161 records, including 2899 from Embase, 1180 from MEDLINE, and 82 from the Cochrane Library. After removal of 862 duplicates, 3299 records remained for title-and-abstract screening. Of these, 3141 records were excluded at the title-and-abstract stage, leaving 158 full-text papers for eligibility assessment. After exclusion of 124 full-text papers, 34 studies were included in the quantitative meta-analysis (Figure 2).

In the additional targeted screening of deployment-relevant sepsis AI studies, several landmark studies were identified, including studies evaluating NAVOY Sepsis, Targeted Real-Time Early Warning System (TREWS), Conformal Multidimensional Prediction of Sepsis Risk (COMPOSER), the Epic Sepsis Model, Sepsis ImmunoScore, and InSight-related approaches. Studies that met the eligibility criteria for prediction-model performance synthesis were included in the evidence table. Studies primarily designed as randomized clinical validation, prospective implementation, quasi-experimental deployment, regulatory validation, or external validation of a commercial model were not automatically pooled with retrospective model-development studies. Instead, they were summarized separately to avoid conflating retrospective discrimination performance with evidence of clinical effectiveness or implementation impact.

### Study Characteristics

Table 1 presents a summary of the selected papers. Of the 34 included studies, 8 (23.53%) were published in 2021, 6 (17.565%) each in 2020, 2022, and 2023, 3 (8.82%) in 2019, 2 (5.88%) in 2024, and 1 (2.94%) each in 2015, 2016, and 2017. In total, 14 of 34 (41.18%) studies used publicly available datasets such as MIMIC, while 20 of 34 (58.82%) used institution-specific inpatient data.

**Table 1.** Summary characteristics of the included studies^a^.

	Study	Dataset family or source	Care setting	Sample size	Sepsis definition	Prediction window (hours)	Validation maturity	AUROC^b^
1	Burdick et al (2020) [28]	Institution-specific; 461 US hospitals	Mixed inpatient or multicenter	20,647	Sepsis-3	48	Retrospective external validation	0.948
2	Barghi and Azadeh-Fard (2022) [29]	Institution-specific teaching hospital	Hospital inpatient; setting NR^c^	20,005	Sepsis-3	24	Retrospective development or internal validation	0.908
3	Mahyoub et al (2023) [30]	Institution-specific hospital EMR^d^	Hospital inpatient; setting NR	17,750	Sepsis-3	6	Retrospective development or internal validation	0.970
4	Kwon et al (2021) [31]	Institution-specific hospital ECG^e^	Hospital-wide inpatient	46,017	Sepsis-3	12	Retrospective development or internal validation	0.906
5	Aşuroğlu and Oğul (2021) [32]	MIMIC-III^f^	ICU^g^	5154	Sepsis-3	12	Retrospective development or internal validation	0.982
6	Gupta et al (2020) [33]	Institution-specific HIPAA database	Hospital inpatient; setting NR	16,909	Sepsis-3	24	Retrospective development or internal validation	0.840
7	Kok et al (2020) [34]	PhysioNet; exact cohort unclear	ICU or critical care dataset	2932	Sepsis-3	6	Retrospective development or internal validation	0.980
8	Barton et al (2019) [35]	MIMIC-III	ICU	91,445	Sepsis-3	24	Retrospective development or internal validation	0.880
9	Al-Mualemi and Lu (2021) [36]	PhysioNet 2019 Challenge or MIMIC-derived	ICU	40,336	Sepsis-3	Unclear	Retrospective development or internal validation	0.952
10	Oei et al (2021) [37]	MIMIC-III	ICU	48,632	Sepsis-3	3	Retrospective development or internal validation	0.850
11	Bedoya et al (2020) [38]	Duke University EHR^h^	Hospital inpatient	42,979	Sepsis-3	5	Retrospective development or internal validation	0.882
12	Lauritsen et al (2020) [39]	Four Danish EHR systems	Multihospital inpatient	3126	Sepsis-3	3	Retrospective development or internal validation	0.856
13	Kam and Kim (2017) [40]	MIMIC-III	ICU^g^	6362	Sepsis-3	2	Retrospective development or internal validation	0.929
14	Ghias et al (2023) [41]	PhysioNet 2019 Challenge	ICU	40,336	Sepsis-3	6	Retrospective development or internal validation	0.980
15	Li et al (2023) [42]	MIMIC-IV	ICU	4603	Sepsis-3	24	Retrospective development or internal validation	0.880
16	Hu et al (2023) [43]	MIMIC-IV	ICU	1167	Sepsis-3	Unclear	Retrospective development or internal validation	0.756
17	Desautels et al (2016) [44]	MIMIC-III	ICU	22,853	Sepsis-3	4	Retrospective development or internal validation	0.880
18	Zhang et al (2022) [45]	MIMIC-IV	ICU	6503	Sepsis-3	Unclear	Retrospective development or internal validation	0.731
19	Scherpf et al (2019) [46]	MIMIC-III	ICU	30,000	Sepsis-3	12	Retrospective development or internal validation	0.810
20	Moor et al (2023) [47]	MIMIC-III, eICU^i^, HiRID^j^, and AUMC^k^	Multicohort ICU	136,478	Sepsis-3	3.7	Retrospective external or multicohort validation	0.846
21	Mollura et al (2021) [48]	MIMIC-III	ICU	142	Sepsis-3	1	Retrospective development or internal validation	0.920
22	Wang and Yao (2022) [49]	PhysioNet 2019 Challenge	ICU	40,336	Sepsis-3	6	Retrospective development or internal validation	0.892
23	Duan et al (2023) [50]	Shanghai ICU infection department	ICU	282	Sepsis-2	6	Retrospective development or internal validation	0.920
24	Delahanty et al (2019) [51]	49 urban community hospitals EHR	Multicenter hospital inpatient	2,759,529	Sepsis-3	24	Retrospective external or multicenter validation	0.970
25	Yuan et al (2020) [52]	Taipei Medical University ICU EMR	ICU	1588	Sepsis-3	Unclear	Prospective observational validation	0.890
26	Kamaleswaran et al (2021) [53]	Institution-specific ICU data	ICU	5748	Sepsis-3	12	Retrospective development or internal validation	0.970
27	Goh et al (2021) [54]	Singapore government hospital EMR	Hospital inpatient	5317	Sepsis-3	48	Retrospective development or internal validation	0.940
28	Steinbach et al (2024) [55]	Leipzig, UMG^l^, and MIMIC-IV	Multicenter mixed inpatient	1,381,358	Sepsis-3	48	Retrospective external or multicenter validation	0.872
29	Persson et al (2024) [56]	Skåne University Hospital ICU	ICU	304	Sepsis-3	3	Prospective randomized clinical validation	0.800
30	Ivanov et al (2022) [57]	16 participating hospitals	Emergency department or triage	615,581	Sepsis-2	6	Retrospective external or multicenter validation	0.942
31	Taneja et al (2021) [58]	Institution-specific EMR	Hospital inpatient	1400	Sepsis-3	12	Prospective observational validation	0.830
32	Henry et al (2015) [59]	MIMIC-II	ICU	13,181	Sepsis-3	28	Retrospective development or internal validation	0.830
33	Henry et al (2022) [60]	TREWS^m^ EHRs	Multisite hospital implementation	469,419	Sepsis-3	3.6	Prospective implementation evaluation	0.970
34	Yu et al (2022) [61]	Barnes-Jewish Hospital or Washington University EHR	Hospital inpatient	70,034	Sepsis-3	6	Retrospective development or internal validation	0.862

a Prediction window indicates the reported lead time before sepsis onset or clinical confirmation. The AUROC values for Al-Mualemi and Lu [36] and Oei et al [37] were standardized to 0.952 and 0.850, respectively, to match the quantitative synthesis and Table S2 in Multimedia Appendix 1.

b AUROC: area under the receiver operating characteristic curve.

c NR: not reported.

d EMR: electronic medical record.

e ECG: electrocardiogram.

f MIMIC: Medical Information Mart for Intensive Care.

g ICU: intensive care unit.

h EHR: electronic health record.

i eICU: eICU Collaborative Research Database.

j HiRID: High Time-Resolution ICU Dataset.

k AUMC: Amsterdam University Medical Center.

l UMG: University Medical Center Göttingen.

m TREWS: Targeted Real-Time Early Warning System.

Sepsis-3 was the most commonly used definition (32/34, 94.12%), with 2 of 34 (5.88%) studies using Sepsis-2. The average number of study participants was 175,543 (SD 525,734), ranging from 142 to 2,759,529 patients. Reported prevalence varied widely (0.15%‐50%), reflecting substantial heterogeneity in definitions, populations, and study settings. Demographic and vital sign data were used in 31 of 34 (91.18%) studies, along with laboratory tests, clinical observations, biomarkers, and blood gas analyses in the majority of studies. The number of predictive variables ranged from 6 to 451.

Regarding model types, random forest was used in 5 of 34 (14.71%) studies, logistic regression in 7 (20.59%), support vector machine in 3 (8.82%), and extreme gradient boosting in 8 (23.53%). Most studies (31/34, 91.18%) used a retrospective design, while 3 of 34 (8.82%) were prospective. All studies used AUROC as the primary outcome metric. Prediction windows were reported heterogeneously across studies, and the “hours before onset” variable in Table 1 refers to the reported lead time before sepsis onset or clinical confirmation where available. Because timing definitions differed across studies, summary averages for prediction timing were not emphasized. Additionally, 21 of 34 (61.76%) studies focused on predicting sepsis within 24 hours of admission. Reported AUROC values ranged from 0.731 to 0.982. These study-level estimates were not interpreted as directly comparable because the studies differed in cohort composition, outcome definition, prediction window, validation design, and dataset independence.

Dataset-provenance review identified repeated use of public cohorts. In total, 1 study used MIMIC-II, 9 included MIMIC-III, and 4 included MIMIC-IV. A total of 3 studies [36,41,49] used the PhysioNet 2019 Challenge dataset and were considered potentially nonindependent. Because several reports used overlapping or related public data sources, the 34 included reports did not represent 34 fully independent patient populations. Complete exclusion of overlap among institution-specific studies was also not possible when study periods or participating institutions were incompletely reported.

Detailed information on dataset provenance, external validation, missing-data handling, baseline SOFA operationalization, and potential cohort overlap is provided in Table S2 in Multimedia Appendix 1.

### Risk-of-Bias and Applicability Assessment

The PROBAST+AI-oriented assessment identified several recurring risk-of-bias and applicability concerns across the included studies. Most studies were retrospective model-development or validation studies, which raised concerns regarding patient selection, outcome labeling, temporal validation, and transportability. Although many studies described the model architecture and input variables, reporting was less consistent for calibration, threshold selection, missing-data mechanisms, imputation strategy, and external validation.

Applicability concerns were also common because the included studies varied substantially in care setting, sepsis definition, data source, prediction window, and measurement frequency. Models developed using single-institution or hospital-specific datasets may have captured local patient characteristics and workflow patterns, but their performance may not generalize to other institutions without external validation. In addition, studies in general wards may be affected by sparse vital sign and laboratory measurement patterns, whereas ICU-based models may not generalize to lower-acuity settings.

Overall, the risk-of-bias and applicability assessment supported a cautious interpretation of the pooled AUROC. The findings suggest that the included models demonstrate potentially useful discrimination under study-specific conditions, but the certainty and generalizability of this evidence are limited by retrospective designs, heterogeneous outcome definitions, incomplete calibration reporting, and limited prospective or external validation. A detailed study-level risk-of-bias and applicability assessment is presented in Table 2.

**Table 2.** PROBAST^a^+AI-oriented risk-of-bias and applicability assessment of included studies^b^.

	Authors	Participants or data source	Predictors	Outcome definition	Analysis or modeling	Missing data	Validation	Calibration or threshold reporting	Overall risk of bias	Applicability concern	Key concern
1	Burdick et al (2020) [28]	Some concerns^c^	Low^d^	Low	Some concerns	Low	High^e^	Some concerns	Some concerns	Some concerns	Retrospective design and limited external validation reporting
2	Barghi and Azadeh-Fard (2022) [29]	Some concerns	Low	Low	Some concerns	Low	High	NR^f^	Some concerns	Some concerns	Limited validation and clinical applicability discussion
3	Mahyoub et al (2023) [30]	Some concerns	Low	Low	Some concerns	Low	High	NR	High	Some concerns	Limited validation, explainability, and applicability reporting
4	Kwon et al (2021) [31]	Some concerns	Some concerns	Low	Some concerns	Low	Low	Some concerns	Some concerns	Some concerns	Retrospective design and limited clinical applicability discussion
5	Aşuroğlu and Oğul (2021) [32]	Some concerns	Some concerns	Low	Some concerns	Low	High	NR	High	Some concerns	Public ICU^g^ dataset and limited external validation
6	Gupta et al (2020) [33]	Some concerns	Some concerns	Low	Some concerns	Low	High	NR	Some concerns	Some concerns	Limited validation and reporting of transportability
7	Kok et al (2020) [34]	Some concerns	Some concerns	Low	Some concerns	Low	High	NR	High	Some concerns	Limited feature transparency and external validation
8	Barton et al (2019) [35]	Some concerns	Low	Low	Some concerns	Low	High	NR	Some concerns	Some concerns	Retrospective design and limited external validation
9	Al-Mualemi and Lu (2021) [36]	Some concerns	Some concerns	Low	Some concerns	Low	High	NR	High	Some concerns	Limited reporting of validation, explainability, and clinical applicability
10	Oei et al (2021) [37]	Some concerns	Low	Low	Some concerns	Low	Low	NR	Some concerns	Some concerns	Limited clinical applicability and calibration reporting
11	Bedoya et al (2020) [38]	Some concerns	Low	Low	Some concerns	Low	High	NR	High	Some concerns	Retrospective single-system evidence and limited external validation
12	Lauritsen et al (2020) [39]	Some concerns	High	Low	Some concerns	High	High	NR	High	High	Incomplete reporting of prevalence, missing data, predictors, and validation
13	Kam and Kim (2017) [40]	Some concerns	Some concerns	Low	Some concerns	Low	High	NR	High	Some concerns	Limited validation and reporting of applicability
14	Ghias et al (2023) [41]	Some concerns	Low	Low	Some concerns	Low	Low	NR	Some concerns	Some concerns	Limited calibration and prospective validation
15	Li et al (2023) [42]	Some concerns	Low	Low	Some concerns	Low	Low	NR	Some concerns	Some concerns	Retrospective design despite relatively complete reporting
16	Hu et al (2023) [43]	Some concerns	Low	Low	Some concerns	Low	Low	NR	Some concerns	Some concerns	Limited prospective validation and calibration reporting
17	Desautels et al (2016) [44]	Some concerns	Low	Low	Some concerns	Low	Low	NR	Some concerns	Some concerns	Retrospective validation and limited calibration reporting
18	Zhang et al (2022) [45]	Some concerns	Low	Low	Some concerns	Low	Low	NR	Some concerns	Some concerns	Incomplete prevalence reporting and limited clinical implementation evidence
19	Scherpf et al (2019) [46]	Some concerns	Low	Low	Some concerns	Low	Low	NR	Some concerns	Some concerns	Incomplete prevalence reporting and retrospective validation
20	Moor et al (2023) [47]	Low	Low	Low	Low	Low	Low	Some concerns	Some concerns	Some concerns	Strong reporting but retrospective design and deployment uncertainty remain
21	Mollura et al (2021) [48]	High	Low	Low	Some concerns	Low	Low	Some concerns	Some concerns	High	Very small sample size and limited generalizability
22	Wang and Yao (2022) [49]	Some concerns	Low	Low	Some concerns	Low	Low	Some concerns	Some concerns	Some concerns	Public challenge dataset and limited implementation evidence
23	Duan et al (2023) [50]	High	Low	Some concerns	Some concerns	Low	Low	NR	Some concerns	High	Small sample size, Sepsis-2 definition, and setting-specific data
24	Delahanty et al (2019) [51]	Low	Low	Low	Some concerns	Low	Low	NR	Some concerns	Some concerns	Retrospective multicenter evidence with limited calibration reporting
25	Yuan et al (2020) [52]	Some concerns	Low	Low	Some concerns	Low	Low	NR	Some concerns	Some concerns	Prospective evidence but limited calibration and broader validation reporting
26	Kamaleswaran et al (2021) [53]	Some concerns	Low	Low	Some concerns	Low	Low	NR	Some concerns	Some concerns	Low event count and limited deployment evidence
27	Goh et al (2021) [54]	Some concerns	Low	Low	Some concerns	Low	Low	NR	Some concerns	Some concerns	Retrospective design and limited prospective implementation evidence
28	Steinbach et al (2024) [55]	Low	Low	Low	Low	Low	Low	Some concerns	Some concerns	Some concerns	Strong reporting but clinical deployment and calibration evidence remain limited
29	Persson et al (2024) [56]	High	Low	Low	Some concerns	Low	Low	NR	Some concerns	Some concerns	Randomized or prospective validation but small sample and implementation context differ
30	Ivanov et al (2022) [57]	Low	Low	Some concerns	Some concerns	Low	Low	Some concerns	Some concerns	Some concerns	Sepsis definition and proprietary implementation context may limit comparability
31	Taneja et al (2021) [58]	Some concerns	Low	Low	Some concerns	Low	High	Some concerns	Some concerns	Some concerns	Prospective or real-world evidence but limited external validation
32	Henry et al (2015) [59]	Some concerns	Low	Low	Some concerns	Low	High	Some concerns	Some concerns	Some concerns	Retrospective development and limited external validation
33	Henry et al (2022) [60]	Low	Low	Low	Some concerns	Low	High	Some concerns	Some concerns	Some concerns	Implementation evidence with alert-response and workflow dependence
34	Yu et al (2022) [61]	Some concerns	Low	Some concerns	Some concerns	Low	High	Some concerns	Some concerns	Some concerns	Retrospective single-system evidence and limited external validation

a PROBAST: Prediction Model Risk of Bias Assessment Tool.

b The assessment was adapted from PROBAST+AI domains and used to guide interpretation rather than to exclude studies.

c Some concerns indicate partial or incomplete reporting or moderate concern.

d Low indicates low concern based on reported information.

e High indicates major concern.

f NR: not reported.

g ICU: intensive care unit.

### Meta-Analysis of Model Performance

Clinical and methodological heterogeneity was extreme across the 34 reports. Under the logit-transformed HKSJ random-effects model, the pooled AUROC was 0.913 (95% CI 0.887‐0.933), with τ^2^=0.665 on the logit-AUROC scale, *I*^2^=99.98%, and a 95% prediction interval of 0.660‐0.983. The prediction interval was substantially wider than the CI around the pooled mean, indicating that the discrimination expected in a new clinical setting could differ considerably from the average estimate.

Accordingly, the overall pooled AUROC was not interpreted as a single generalizable estimate of sepsis prediction performance. It represents an average across studies that varied in care setting, data source, sepsis definition, prediction horizon, measurement frequency, model architecture, and validation design. The lower bound of the prediction interval suggests that some models implemented in new populations may achieve only modest discrimination despite a relatively high pooled mean.

The primary report-level analysis may also have been affected by nonindependence because multiple studies reused MIMIC- or PhysioNet-derived cohorts. In the dataset-family sensitivity analysis, one prespecified representative report was retained for each overlapping public dataset family, while studies using institution-specific or otherwise independent datasets were retained. This analysis included 22 reports and yielded a pooled AUROC of 0.923 (95% CI 0.893‐0.945), with τ^2^=0.643 on the logit-AUROC scale, *I*^2^=99.98%, and a 95% prediction interval of 0.683‐0.985. Compared with the primary analysis, the pooled AUROC increased slightly from 0.913 to 0.923, while the CI became modestly wider, and the prediction interval remained broad. These findings indicate that repeated use of public datasets did not materially alter the overall average estimate, but substantial between-study heterogeneity and uncertainty regarding performance in new clinical populations persisted after accounting for dataset overlap.

Threshold-specific sensitivity and specificity were not reported sufficiently consistently to support a representative bivariate or hierarchical summary receiver operating characteristic analysis. The present synthesis therefore summarizes discrimination rather than performance at a common clinical decision threshold.

**Figure 3.**
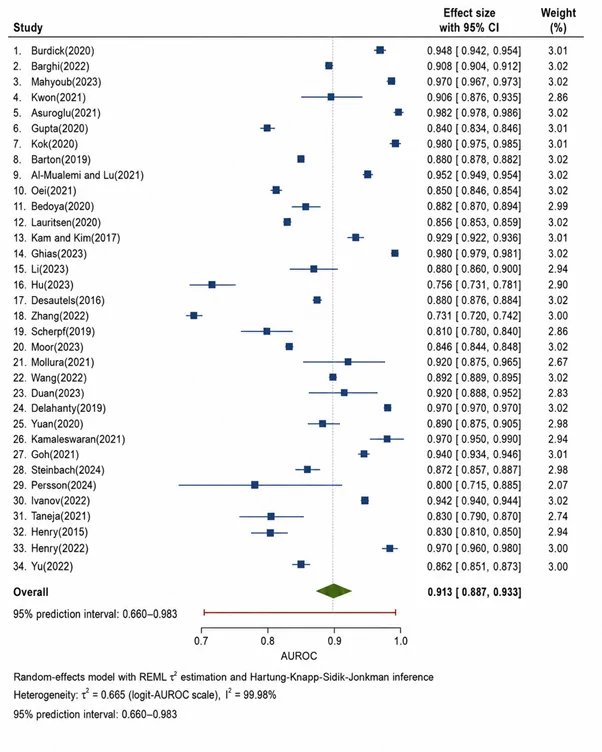
Forest plot of study-specific AUROC estimates and Hartung-Knapp-Sidik-Jonkman random-effects summary for AI-based sepsis prediction models in hospitalized adults. The diamond indicates the pooled AUROC, and the horizontal prediction interval indicates the expected range of underlying model performance in a comparable new clinical setting [28-61]. AUROC: area under the receiver operating characteristic curve; REML: restricted maximum likelihood.

In an exploratory assessment of reporting bias, Deeks funnel plot asymmetry testing among 9 studies with sufficient threshold-specific diagnostic-accuracy data did not indicate significant funnel plot asymmetry or small-study effects (*P*=.17; Figure S5 in Multimedia Appendix 1). The study-specific AUROC estimates and the HKSJ random-effects summary are presented in Figure 3.

### Subgroup and Evidence-Tier Analyses

In the exploratory prediction-window analysis, 8 studies evaluating models with lead times of up to 4 hours had a pooled AUROC of 0.894 (95% CI 0.829‐0.936; 95% prediction interval 0.629‐0.977). The 22 studies evaluating prediction more than 4 hours before sepsis onset or clinical confirmation had a pooled AUROC of 0.926 (95% CI 0.897‐0.948; 95% prediction interval 0.685‐0.986). In total, 4 studies with unclear or unreported prediction windows had a pooled AUROC of 0.858 (95% CI 0.581‐0.964; 95% prediction interval 0.183‐0.994). Although the average AUROC was higher in the more-than-4-hour subgroup than in the within-4-hour subgroup, the CIs and prediction intervals overlapped substantially. These findings do not establish the clinical superiority of either prediction horizon. Prediction-window definitions varied across studies, and the 4-hour cut point was selected post hoc rather than prespecified in the PROSPERO protocol; therefore, these subgroup findings should be interpreted as exploratory. The prediction-window subgroup analysis is presented in Figure S4 in Multimedia Appendix 1; additional exploratory subgroup analyses by publication year, dataset, and sample size are presented in Figures S1‐S3 in Multimedia Appendix 1.

To avoid conflating different levels of evidence, studies were also interpreted according to validation maturity and implementation design, as summarized in Table S3 in Multimedia Appendix 1. Most studies contributing to the quantitative AUROC synthesis were retrospective model-development or internal-validation studies. These studies provided evidence of discrimination within development or closely related validation samples but did not establish transportability or clinical effectiveness. Retrospective external-validation studies provided more direct information on transportability, although performance varied across institutions and model versions. Prospective implementation, quasi-experimental deployment, regulatory validation, and randomized clinical studies were fewer and evaluated different outcomes, including alert response, treatment processes, workflow integration, and mortality. These studies were therefore summarized narratively rather than pooled with retrospective AUROC studies. Taken together, the validation-maturity assessment indicated that high retrospective discrimination did not consistently translate into improved clinical outcomes; model performance, calibration, alert burden, clinician response, and implementation fidelity should therefore be evaluated separately.

### Contextual Description of Sepsis-Related Health Care Burden

The Korean claims analysis was conducted separately from the systematic review and meta-analysis to provide contextual information on sepsis-related health care use. No AI model was developed, applied, or validated using the HIRA-NIS data. Table 3 presents unadjusted descriptive characteristics by sepsis-related episode timing.

**Table 3.** Descriptive summary of patient characteristics and health care use by sepsis-related episode group and observation period^a^.

Group and variable	≤1 Month			>1 Month		
	Values, n	Mean (SD)	Median (IQR)	Values, n	Mean (SD)	Median (IQR)
General inpatient episodes	851,190			82,883		
Age (years)		57.8 (17.7)	55.0 (45.0-65.0)		69.1 (17.1)	65.0 (55.0-90.0)
LoS^b^ (days)		6.0 (6.2)	4.0 (2.0-8.0)		130.4 (123.9)	63.0 (36.0-194.0)
Cost (KRW)^c^		2,227,457 (3,115,725)	1,293,000 (610,000-2,591,000)		15,765,971 (16,514,854)	11,137,000 (4,579,000-23,667,000)
Surgeries, n		0 (1)	0 (0-1)		0 (1)	—^d^
Sepsis episodes	66			72		
Age (years)		80.2 (12.2)	90.0 (75.0-90.0)		78.7 (14.3)	90.0 (65.0-90.0)
LoS (days)		13.9 (7.5)	12.5 (7.0-19.0)		96.3 (74.4)	66.0 (44.5-120.5)
Cost (KRW)		4,218,364 (4,689,154)	2,462,000 (1,353,000-5,486,000)		13,847,236 (10,794,240)	10,781,000 (5,639,000-18,300,500)
Surgeries, n		0 (0)	—		1 (1)	0 (0-1)
Prior sepsis followed by subsequent inpatient episode	460			282		
Age (years)		77.8 (13.9)	75.0 (65.0-90.0)		78.0 (13.2)	75.0 (75.0-90.0)
LoS (days)		11.1 (8.2)	9.0 (4.0-17.0)		105.4 (90.6)	66.5 (42.0-132.0)
Cost (KRW)		4,679,144 (5,084,302)	3,277,500 (1,394,500-6,304,000)		16,265,837 (14,595,386)	12,204,500 (6,785,000-20,643,000)
Surgeries, n		0 (1)	0 (0-1)		0 (1)	0 (0-1)
Inpatient episode followed by subsequent sepsis	1969			2971		
Age (years)		78.5 (13.7)	75.0 (75.0-90.0)		80.2 (12.4)	90.0 (75.0-90.0)
LoS (days)		14.2 (8.0)	14.0 (8.0-20.0)		149.1 (99.6)	122.0 (59.0-227.0)
Cost (KRW)		4,824,147 (5,884,546)	2,818,000 (1,639,000-5,998,000)		17,238,988 (12,462,788)	14,582,000 (7,667,000-23,776,000)
Surgeries, n		0 (1)	0 (0-1)		0 (1)	—

a General inpatient episodes refer to eligible inpatient episodes without sepsis-related classification in the analytic window. Sepsis episodes refer to inpatient episodes with sepsis identified using *International Classification of Diseases* (ICD) primary diagnosis codes A40.0-A40.3, A40.8-A40.9, A41.0-A41.5, and A41.8-A41.9. Prior sepsis followed by subsequent inpatient episode refers to patients with a sepsis episode followed by a later inpatient episode within the defined observation window. Inpatient episode followed by subsequent sepsis refers to patients with an inpatient episode followed by a later sepsis episode within the defined observation window.

b LoS: length of stay.

c Total medical cost is presented in Korean won (KRW). The exchange rate was KRW 1411.51=US $1 as of August 14, 2026.

d Not available.

Compared with general inpatient episodes, sepsis episodes showed longer mean length of stay and higher total medical costs. In the early observation window, sepsis episodes had a mean length of stay of 13.9 (SD 7.5) days and mean total medical cost of KRW 4,218,364 (SD KRW 4,689,154; KRW 1411.51=US $1 as of August 14, 2026), compared with 6.0 (SD 6.2) days and KRW 2,227,457 (SD KRW 3,115,725) among general inpatient episodes. Inpatient episodes followed by subsequent sepsis also showed elevated health care use, with a mean length of stay of 14.2 (SD 8.0) days and mean total medical cost of KRW 4,824,147 (SD KRW 5,884,546). The HIRA-NIS findings are presented descriptively without formal hypothesis testing because the episode groups differed substantially in sample size, age distribution, and likely clinical severity.

Sepsis-related episode groups showed longer observed lengths of stay and higher unadjusted medical costs than general inpatient episodes. However, these groups also differed markedly in age and likely differed in comorbidity and illness severity. The results therefore describe observed burden patterns and should not be interpreted as adjusted associations or causal effects of sepsis timing.

## Discussion

### Principal Findings

This systematic review found that AI-based sepsis prediction models often achieved good discrimination within individual study settings [62-64], but the pooled average concealed substantial variation across studies [65-67]. Using a logit-transformed HKSJ random-effects model, the overall pooled AUROC was 0.913 (95% CI 0.887‐0.933), whereas the 95% prediction interval ranged from 0.660 to 0.983. The substantially wider prediction interval indicates that performance in a comparable new clinical population could vary from modest to very high and is therefore more informative for clinical interpretation than the CI around the pooled mean alone.

A key advantage of ML and DL models lies in their ability to detect subtle physiological deviations that may signal the onset of sepsis before clinical recognition. Traditional systems—such as the Systemic Inflammatory Response Syndrome, quick SOFA, and National Early Warning Score—have been widely used for early sepsis identification [20,28,62,65,68-70]. However, these rule-based tools often show limited sensitivity and specificity across heterogeneous inpatient populations [28,36,40]. In contrast, AI models can integrate multidimensional data, including vital signs, laboratory results, and patient histories, to identify complex and nonlinear risk patterns that may not be captured by conventional approaches [28,69,71]. Nevertheless, strong retrospective discrimination should not be interpreted as evidence of transportability or clinical effectiveness without external and prospective validation.

Repeated use of public datasets also reduced the independence of the evidence base. After potentially overlapping reports were reduced to one prespecified representative report per dataset family, the pooled AUROC changed only slightly from 0.913 to 0.923. However, the 95% prediction interval remained wide (0.683‐0.985), indicating that dataset overlap did not fully explain the substantial variability in model performance across clinical settings. These findings suggest that differences in patient populations, care settings, outcome definitions, prediction windows, and validation designs remained important sources of heterogeneity.

### Comparison With Prior Work

Previous studies have consistently shown that AI-based sepsis prediction models can achieve favorable discrimination within their original development or validation settings, but reported performance varies substantially according to data composition, feature selection, model architecture, prediction horizon, and validation strategy [67]. Our findings are consistent with this broader literature, while extending it by emphasizing prediction intervals, dataset dependence, and validation maturity rather than relying primarily on a single pooled AUROC.

Models developed using hospital-specific datasets have sometimes shown stronger discrimination than models evaluated using public databases such as MIMIC [72]. Local datasets may better reflect institution-specific patient characteristics, measurement practices, and clinical workflows. However, stronger performance within a local dataset may also reflect overfitting or dependence on site-specific patterns and should not be interpreted as evidence of broad transportability. Independent external validation remains necessary before localized performance can be generalized to other hospitals or care settings [72].

Sample size also significantly influenced model stability and performance. Studies using larger datasets reported more consistent and higher AUROC values, supporting the need for scalable and robust clinical data infrastructures [73,74]. Nevertheless, sample size alone does not ensure generalizability. Models may still perform inconsistently when transported across populations that differ in case mix, outcome labeling, predictor availability, missing-data mechanisms, and validation design. The wide prediction interval observed in this review similarly indicates that a high pooled mean does not guarantee comparable performance in a new hospital.

The interpretation of prediction timing also differs from that of earlier analyses that emphasized short-horizon performance. In the present HKSJ analysis, models predicting sepsis more than 4 hours before onset had a higher average pooled AUROC than models predicting within 4 hours [75-77], although their CIs and prediction intervals overlapped substantially. Therefore, these findings do not establish the superiority of either prediction horizon. Higher discrimination close to sepsis onset may reflect stronger physiological signals, as clinical deterioration becomes more apparent, whereas longer-horizon models may offer more time for diagnostic evaluation and treatment preparation. Clinical usefulness consequently depends not only on AUROC but also on whether alerts are generated early enough to support clinician review, diagnostic confirmation, and timely initiation of care [72,73,78].

The distinction between retrospective model performance and prospective clinical implementation is particularly important. The NAVOY Sepsis randomized clinical validation study, the TREWS prospective multisite implementation study, and the COMPOSER quasi-experimental deployment study provide clinically relevant evidence but address different questions from retrospective AUROC-based model development [56,79,80]. NAVOY Sepsis did not demonstrate a consistent mortality benefit in the alerted group, whereas TREWS and COMPOSER suggested that clinician response and workflow integration may influence treatment processes and outcomes. These findings indicate that discrimination alone is insufficient to determine clinical effectiveness.

Similarly, the Sepsis ImmunoScore study provides regulatory and validation evidence for a US Food and Drug Administration–authorized AI- and ML-based sepsis tool [81]. In contrast, external evaluations of the Epic Sepsis Model, including Wong et al [82,83] and a subsequent multicenter prospective validation of version 2 of the Epic Sepsis Model published during paper revision, demonstrated that discrimination, calibration, transportability, and alert burden may vary across institutions. Development performance therefore cannot be assumed to transfer directly to routine clinical practice.

This review also extends previous work by including studies conducted across general wards, EDs, and ICUs rather than focusing solely on critical care populations. This broader scope increases the relevance of the review across hospitalized adults but also introduces additional heterogeneity. Monitoring frequency, laboratory availability, missing-data patterns, illness severity, and clinical workflows differ markedly across care settings [73,74,84]. AI models intended for broad deployment should therefore undergo external validation across multiple care environments and should be assessed for calibration, alert burden, clinician adoption, and patient outcome effects.

The very high heterogeneity observed in the AUROC synthesis further limits interpretation of a single pooled estimate. Although AUROC was the most consistently reported performance metric, threshold-specific sensitivity, specificity, and 2×2 diagnostic accuracy data were incompletely reported. This prevented a representative bivariate or hierarchical summary receiver operating characteristic synthesis. Accordingly, the pooled AUROC should be interpreted as a descriptive summary of discrimination rather than evidence of diagnostic accuracy at a common clinical threshold, clinical effectiveness, or readiness for deployment.

The Korean HIRA-NIS analysis was analytically separate from the model-performance synthesis. Sepsis-related episode groups showed longer observed hospital stays and higher unadjusted medical costs than general inpatient episodes. These findings provide contextual information on the clinical and economic burden of sepsis-related episodes but do not validate an AI model or demonstrate that AI-based early warning systems reduce use, costs, or mortality.

For clinical implementation, discrimination should be evaluated alongside calibration, false-positive frequency, alert burden, prediction lead time, clinician response, workflow integration, and patient outcome effects. Most included studies evaluated retrospective discrimination rather than prospective workflow impact or clinical outcomes [75,76,85]. Therefore, the findings do not support broad deployment based on AUROC alone. Future studies should determine whether alerts provide sufficiently timely, accurate, interpretable, and actionable information for clinicians.

Policy decisions should likewise not rely solely on retrospective model-performance estimates. Interoperable data infrastructure, transparent reporting standards, independent external validation, and prospective postdeployment monitoring are needed before AI-based sepsis prediction systems are adopted routinely across heterogeneous hospital settings. Ethical and equity considerations should also be incorporated because model performance and data quality may vary by age, sex, socioeconomic status, race or ethnicity, and care setting. Future evaluations should assess subgroup performance, fairness, and transportability to reduce the risk of underperformance in underrepresented populations.

### Limitations

First, the included studies were clinically and methodologically heterogeneous. They differed in sepsis definition, baseline SOFA operationalization, care setting, case mix, prediction horizon, predictor measurement frequency, missing-data handling, and validation design. Although subgroup analyses were conducted, residual heterogeneity remained substantial.

Second, several reports used overlapping MIMIC or PhysioNet-derived cohorts. These studies evaluated different model architectures but were not statistically independent at the patient level. The primary report-level analysis may therefore overrepresent selected public datasets and underestimate uncertainty. Dataset-family sensitivity analyses reduced but could not eliminate this concern because some cohort relationships and sampling periods were incompletely reported.

Third, study-level AUROC uncertainty was incompletely reported. SEs had to be derived from published CIs where possible, and some studies did not provide sufficiently precise variance information. Although the HKSJ approach and prediction intervals were used to provide more conservative inference, the pooled results remain dependent on the quality of the reported study-level estimates.

Fourth, AUROC does not directly indicate calibration, clinical threshold performance, positive predictive value, or alert burden. A bivariate or hierarchical summary receiver operating characteristic analysis could not be performed representatively because threshold-specific sensitivity, specificity, and 2×2 data were unavailable for many studies.

Fifth, the prediction-window analysis was exploratory and used a post hoc 4-hour cut point. Timing definitions were not standardized, and some studies defined prediction relative to sepsis labels that may have been constructed retrospectively. Higher discrimination closer to onset may therefore partly reflect easier detection rather than more useful early prediction.

Sixth, prospective implementation and randomized studies were too few and too heterogeneous in intervention design and outcomes to support quantitative pooling. Clinical-effectiveness conclusions therefore cannot be drawn from the retrospective discrimination literature.

Seventh, the exploratory Deeks funnel plot asymmetry test was limited to 9 studies with sufficient threshold-specific diagnostic-accuracy data. The absence of detected asymmetry does not exclude publication bias, selective outcome reporting, or preferential publication of high-performing models.

Finally, the HIRA-NIS analysis was descriptive and unadjusted. The episode groups differed substantially in age and likely differed in comorbidity, disease severity, and treatment intensity. These findings should therefore be interpreted only as contextual burden patterns and not as causal estimates or evidence of AI-related benefit.

### Conclusions

AI-based sepsis prediction models frequently demonstrated good discrimination within individual study settings, but their expected performance in new clinical populations remains uncertain. The wide prediction interval, extreme between-study heterogeneity, overlapping public datasets, and predominance of retrospective studies limit the generalizability of the overall pooled AUROC. Prediction-window and validation-maturity analyses provide more clinically meaningful information than a single average performance estimate, although these subgroup findings remain exploratory.

The Korean claims analysis separately documented higher observed health care use among sepsis-related episode groups but did not evaluate or validate an AI model. Before routine clinical implementation, sepsis prediction systems require independent external validation, calibration assessment, transparent reporting of alert thresholds and lead times, prospective workflow evaluation, and randomized or quasi-experimental assessment of patient and process outcomes.

## Supplementary material

10.2196/95665 Multimedia Appendix 1 Detailed search queries and supplementary meta-analysis figures for subgroup meta-analyses by publication year, dataset type, sample size, and prediction timing.

10.2196/95665 Checklist 1 PRISMA 2020 checklist.

## Abbreviations

AUROC: area under the receiver operating characteristic curve
COMPOSER: Conformal Multidimensional Prediction of Sepsis Risk
DL: deep learning
ED: emergency department
HIRA-NIS: National Inpatient Sample from the Health Insurance Review and Assessment Service of Korea
HKSJ: Hartung-Knapp-Sidik-Jonkman
ICD: International Classification of Diseases
ICU: intensive care unit
MIMIC: Medical Information Mart for Intensive Care
ML: machine learning
PRISMA: Preferred Reporting Items for Systematic Reviews and Meta-Analyses
PROBAST: Prediction Model Risk of Bias Assessment Tool
SOFA: Sequential Organ Failure Assessment
TREWS: Targeted Real-Time Early Warning System
