# Supplementary material for: AI-Based Sepsis Prediction in Hospitalized Adults: Systematic Review, Subgroup Meta-Analysis, and Contextual Analysis of Clinical Burden
Source: J Med Internet Res. 2026 Sep 17;28:e95665. doi: 10.2196/95665 (PMC13585307; doi:10.2196/95665)
Supplement: Supplementary file 2 [file jmir-v28-e95665-s001.docx]

**Table S1.** Detailed Search Queries

Embase.com History March 12, 2025

| Search | Embase Query – March 12, 2025 | Items found |
| --- | --- | --- |
| 1 | Artificial intelligence.mp. or exp Artificial intelligence/ | 111,999 |
| 2 | Machine learning.mp. or exp Machine learning/ | 488,791 |
| 3 | Deep learning.mp. or exp Deep learning/ | 78,690 |
| 4 | 1 or 2 or 3 | 538,462 |
| 5 | Sepsis*.mp. or exp Sepsis/ | 397,236 |
| 6 | Septic.mp. or exp shock, Septic/ | 133,987 |
| 7 | 5 or 6 | 426,244 |
| 8 | 4 and 7 | 2,899 |

MEDLINE.com History March 12, 2025

| Search | MEDLINE Query – March 12, 2025 | Items found |
| --- | --- | --- |
| 1 | Artificial intelligence.mp. or exp Artificial intelligence/ | 218,356 |
| 2 | Machine learning.mp. or exp Machine learning/ | 141,894 |
| 3 | Deep learning.mp. or exp Deep learning/ | 65,275 |
| 4 | 1 or 2 or 3 | 301,585 |
| 5 | Sepsis*.mp. or exp Sepsis/ | 218,974 |
| 6 | Septic.mp. or exp shock, Septic/ | 80,066 |
| 7 | 5 or 6 | 249,518 |
| 8 | 4 and 7 | 1,180 |

Cochrane.com History March 12, 2025

| Search | Cochrane Query – March 12, 2025 | Items found |
| --- | --- | --- |
| #1 | (Artificial intelligence):ti,ab,kw (Word variations have been searched) | 2,189 |
| #2 | MeSH descriptor: [Artificial Intelligence] explode all trees | 3,184 |
| #3 | (Machine learning):ti,ab,kw (Word variations have been searched) | 3,076 |
| #4 | MeSH descriptor: [Machine Learning] explode all trees | 983 |
| #5 | (Deep learning):ti,ab,kw (Word variations have been searched) | 1,701 |
| #6 | MeSH descriptor: [Deep Learning] explode all trees | 321 |
| #7 | #1 OR #2 OR #3 OR #4 OR #5 OR #6 | 7,717 |
| #8 | (Sepsis):ti,ab,kw (Word variations have been searched) | 14,135 |
| #9 | MeSH descriptor: [Sepsis] explode all trees | 6,508 |
| #10 | (Septic):ti,ab,kw (Word variations have been searched) | 5,780 |
| #11 | MeSH descriptor: [Shock, Septic] explode all trees | 1,419 |
| #12 | #8 OR #9 OR #10 OR #11 | 18,954 |
| #13 | #7 AND #12 | 82 |

Supplementary Search for Deployment-Relevant and Landmark Sepsis AI Studies

To address the possibility that implementation-focused sepsis AI studies were not captured by generic AI/ML search terms alone, supplementary searches were conducted in Web of Science Core Collection, Scopus, IEEE Xplore, and medRxiv. The search combined sepsis-related terms with AI/ML, early warning, validation, and implementation terms.

Search concept:

(“sepsis” OR “septic shock”) AND (“machine learning” OR “deep learning” OR “artificial intelligence” OR “early warning” OR “clinical deterioration” OR “decision support” OR “real-time alert” OR “sepsis alert” OR “external validation” OR “prospective validation” OR “randomized clinical validation” OR “implementation” OR “deployment”)

Targeted landmark terms:

“TREWS” OR “Targeted Real-Time Early Warning System” OR “Epic Sepsis Model” OR “NAVOY” OR “COMPOSER” OR “Sepsis ImmunoScore” OR “InSight”

Supplementary search results:

Web of Science Core Collection: 10,969 records

Scopus: 10,850 records

IEEE Xplore: 521 records

medRxiv: 1,050 records

Total: 23,390 records

Handling rule:

Records identified through supplementary searches were assessed using the same eligibility criteria as the main review. No additional study was added to the quantitative AUROC meta-analysis. Studies focused on randomized clinical validation, prospective implementation, quasi-experimental deployment, regulatory validation, or external validation of proprietary or commercial models were retained for narrative synthesis when they were not suitable for pooling with retrospective model-development studies.

**Table S2**. Detailed Evidence Characteristics of Included Studies

| **Study** | **Dataset family/source** | **Care setting** | **Sample size** | **Sepsis definition** | **Prediction window** | **Validation maturity** | **External validation** | **Missing-data handling** | **Baseline SOFA handling** | **Cohort overlap flag** | **AUROC** |
| --- | --- | --- | --- | --- | --- | --- | --- | --- | --- | --- | --- |
| Burdick et al (2020) | Institution-specific; 461 US hospitals | Mixed inpatient/multicenter | 20,647 | Sepsis-3 | 48 h | Retrospective external validation | Yes | NR | NR | No | 0.948 |
| Barghi et al (2022) | Institution-specific teaching hospital | Hospital inpatient; setting NR | 20,005 | Sepsis-3 | 24 h | Retrospective development/internal validation | No/unclear | NR | NR | No | 0.908 |
| Mahyoub et al (2023) | Institution-specific hospital EMR | Hospital inpatient; setting NR | 17,750 | Sepsis-3 | 6 h | Retrospective development/internal validation | No/unclear | NR | NR | No | 0.970 |
| Kwon et al (2021) | Institution-specific hospital ECG | Hospital-wide inpatient | 46,017 | Sepsis-3 | 12 h | Retrospective development/internal validation | No/unclear | NR | NR | No | 0.906 |
| Asuroglu et al (2021) | MIMIC-III | ICU | 5,154 | Sepsis-3 | 12 h | Retrospective development/internal validation | No | NR | NR | Yes: MIMIC-III | 0.982 |
| Gupta et al (2019) | Institution-specific HIPAA database | Hospital inpatient; setting NR | 16,909 | Sepsis-3 | 24 h | Retrospective development/internal validation | No/unclear | NR | NR | No | 0.840 |
| Kok et al (2020) | PhysioNet; exact cohort unclear | ICU/critical care dataset | 2,932 | Sepsis-3 | 6 h | Retrospective development/internal validation | No | NR | NR | Possible: PhysioNet | 0.980 |
| Barton et al (2019) | MIMIC-III | ICU | 91,445 | Sepsis-3 | 24 h | Retrospective development/internal validation | No | NR | NR | Yes: MIMIC-III | 0.880 |
| Al-Mualemi and Lu (2021) | PhysioNet 2019 Challenge / MIMIC-derived | ICU | 40,336 | Sepsis-3 | Unclear | Retrospective development/internal validation | No | NR | NR | Yes: PhysioNet 2019 | 0.952 |
| Oei et al (2021) | MIMIC-III | ICU | 48,632 | Sepsis-3 | 3 h | Retrospective development/internal validation | No | NR | NR | Yes: MIMIC-III | 0.850 |
| Bedoya et al (2020) | Duke University EHR | Hospital inpatient | 42,979 | Sepsis-3 | 5 h | Retrospective development/internal validation | No | NR | NR | No | 0.882 |
| Lauritsen et al (2020) | Four Danish EHR systems | Multihospital inpatient | 3,126 | Sepsis-3 | 3 h | Retrospective development/internal validation | Unclear | NR | NR | No | 0.856 |
| Kam and Kim (2017) | MIMIC-III | ICU | 6,362 | Sepsis-3 | 2 h | Retrospective development/internal validation | No | NR | NR | Yes: MIMIC-III | 0.929 |
| Ghias et al (2022) | PhysioNet 2019 Challenge | ICU | 40,336 | Sepsis-3 | 6 h | Retrospective development/internal validation | No | SMOTE reported; other handling NR | NR | Yes: PhysioNet 2019 | 0.980 |
| Li et al (2023) | MIMIC-IV | ICU | 4,603 | Sepsis-3 | 24 h | Retrospective development/internal validation | No | NR | NR | Yes: MIMIC-IV | 0.880 |
| Hu et al (2023) | MIMIC-IV | ICU | 1,167 | Sepsis-3 | Unclear | Retrospective development/internal validation | No | NR | NR | Yes: MIMIC-IV | 0.756 |
| Desautels et al (2016) | MIMIC-III | ICU | 22,853 | Sepsis-3 | 4 h | Retrospective development/internal validation | No | NR | NR | Yes: MIMIC-III | 0.880 |
| Zhang et al (2022) | MIMIC-IV | ICU | 6,503 | Sepsis-3 | Unclear | Retrospective development/internal validation | No | NR | NR | Yes: MIMIC-IV | 0.731 |
| Scherpf et al (2019) | MIMIC-III | ICU | 30,000 | Sepsis-3 | 12 h | Retrospective development/internal validation | No | NR | NR | Yes: MIMIC-III | 0.810 |
| Moor et al (2023) | MIMIC-III, eICU, HiRID, AUMC | Multicohort ICU | 136,478 | Sepsis-3 | 3.7 h | Retrospective external/multicohort validation | Yes | NR | NR | Yes: MIMIC-III; mixed sources | 0.846 |
| Mollura et al (2021) | MIMIC-III | ICU | 142 | Sepsis-3 | 1 h | Retrospective development/internal validation | No | NR | NR | Yes: MIMIC-III | 0.920 |
| Wang et al (2021) | PhysioNet 2019 Challenge | ICU | 40,336 | Sepsis-3 | 6 h | Retrospective development/internal validation | No | NR | NR | Yes: PhysioNet 2019 | 0.892 |
| Duan et al (2023) | Shanghai ICU infection department | ICU | 282 | Sepsis-2 | 6 h | Retrospective development/internal validation | No | NR | Not applicable | No | 0.920 |
| Delahanty et al (2019) | 49 urban community hospitals EHR | Multicenter hospital inpatient | 2,759,529 | Sepsis-3 | 24 h | Retrospective external/multicenter validation | Yes | NR | NR | No | 0.970 |
| Yuan et al (2020) | Taipei Medical University ICU EMR | ICU | 1,588 | Sepsis-3 | Unclear | Prospective observational validation | No/unclear | NR | NR | No | 0.890 |
| Kamaleswaran et al (2020) | Institution-specific ICU data | ICU | 5,748 | Sepsis-3 | 12 h | Retrospective development/internal validation | No | NR | NR | No | 0.970 |
| Goh et al (2021) | Singapore government hospital EMR | Hospital inpatient | 5,317 | Sepsis-3 | 48 h | Retrospective development/internal validation | No/unclear | NR | NR | No | 0.940 |
| Steinbach et al (2024) | Leipzig, UMG, and MIMIC-IV | Multicenter mixed inpatient | 1,381,358 | Sepsis-3 | 48 h | Retrospective external/multicenter validation | Yes | NR | NR | Yes: MIMIC-IV; mixed sources | 0.872 |
| Persson et al (2024) | Skåne University Hospital ICU | ICU | 304 | Sepsis-3 | 3 h | Prospective randomized clinical validation | Yes, prospective | NR | NR | No | 0.800 |
| Ivanov et al (2022) | 16 participating hospitals | Emergency department/triage | 615,581 | Sepsis-2 | 6 h | Retrospective external/multicenter validation | Yes | NR | Not applicable | No | 0.942 |
| Taneja et al (2020) | Institution-specific EMR | Hospital inpatient | 1,400 | Sepsis-3 | 12 h | Prospective observational validation | No/unclear | NR | NR | No | 0.830 |
| Henry et al (2015) | MIMIC-II | ICU | 13,181 | Sepsis-3 | 28 h | Retrospective development/internal validation | No | NR | NR | Yes: MIMIC-II | 0.830 |
| Henry et al (2022) | TREWS EHRs | Multisite hospital implementation | 469,419 | Sepsis-3 | 3.6 h | Prospective implementation evaluation | Yes, multisite | NR | NR | No | 0.970 |
| Yu et al (2022) | Barnes-Jewish Hospital/Washington University EHR | Hospital inpatient | 70,034 | Sepsis-3 | 6 h | Retrospective development/internal validation | No | NR | NR | No | 0.862 |

**Abbreviations**: AUROC, area under the receiver operating characteristic curve; ICU, intensive care unit; MIMIC, Medical Information Mart for Intensive Care; NR, not reported in the extracted manuscript data; SOFA, Sequential Organ Failure Assessment. Cohort-overlap flags identify repeated use of the same public dataset family and do not imply duplicate publications. Care setting and validation maturity were classified from the dataset and study descriptions available in the manuscript. Where the original extraction did not provide a specific missing-data method or baseline SOFA operationalization, the item was recorded as NR rather than inferred. The 34 included studies correspond to references 1-34.

**Figure S1.** Publication-year subgroup meta-analysis of AI-based sepsis prediction models

**Caption**: References for the studies shown in this figure are provided in references 1-34.

**Figure S2.** Dataset subgroup meta-analysis of AI-based sepsis prediction models

**Caption**: References for the studies shown in this figure are provided in references 1-34.

**Figure S3.** Sample-size subgroup meta-analysis of AI-based sepsis prediction models

**Caption**: References for the studies shown in this figure are provided in references 1-34.

**Figure S4.** Prediction-window subgroup meta-analysis of AI-based sepsis prediction models

**Caption**: References for the studies shown in this figure are provided in references 1-34.

**Table S3.** Evidence tier classification

| **Evidence tier** | **Study examples** | **Interpretation** |
| --- | --- | --- |
| Randomized clinical validation | NAVOY Sepsis | Clinical validation evidence; not pooled with retrospective AUROC studies |
| Prospective implementation | TREWS | Workflow and clinician-response evidence |
| Quasi-experimental deployment | COMPOSER | Deployment impact evidence |
| Regulatory/AI tool validation | Sepsis ImmunoScore | Regulatory maturity and validation evidence |
| Proprietary external validation | Epic Sepsis Model, ESM v2 | Transportability, calibration, and alert-burden evidence |
| Retrospective model-development/validation | 34 pooled studies | Quantitative AUROC meta-analysis |

**Note. References:** NAVOY Sepsis [29], TREWS [33], COMPOSER [35], Sepsis ImmunoScore [36], Epic Sepsis Model [37], ESM v2 [38]

**Table S4.** AI-specific PRISMA reporting addendum for systematic reviews of AI/ML prediction models

| **AI-specific reporting item** | **Description** | **Location where addressed** |
| --- | --- | --- |
| AI/ML focus identified in title and abstract | The title and abstract identify the review as a systematic review and meta-analysis of AI/ML-based sepsis prediction models. | Title; Abstract |
| Clinical task and intended use | The manuscript specifies that the target task is prediction or early recognition of sepsis in hospitalized adult patients and distinguishes model performance from clinical implementation. | Introduction; Methods > Study Design; Discussion |
| Care setting and target population | The review reports inpatient adult populations and distinguishes ICU, ED, and general-ward settings where available. | Methods > Eligibility Criteria; Results > Study Characteristics; Table 1 |
| Model type and algorithm reporting | Extracted model types include ML/DL algorithms such as logistic regression, random forest, XGBoost, neural networks, recurrent neural networks, and other architectures where reported. | Methods > Data Extraction; Results > Study Characteristics; Table 1 |
| Data source and development context | The review extracts whether models used public datasets, hospital-specific datasets, multicenter data, or proprietary/commercial systems. | Methods > Data Extraction; Results > Study Characteristics; Table 1 |
| Predictor and feature information | The review extracts input variable categories, including demographics, vital signs, laboratory values, biomarkers, observations, clinical notes, and other structured or unstructured data where reported. | Methods > Data Extraction; Results > Study Characteristics; Table 1 |
| Outcome definition and label construction | Sepsis definition, including Sepsis-2 or Sepsis-3 where reported, was extracted and discussed as a source of heterogeneity. | Methods > Data Extraction; Results > Study Characteristics; Discussion > Limitations |
| Prediction window and timing | Prediction window or hours before onset were extracted and analyzed as subgroup variables where available. | Methods > Data Extraction; Results > Subgroup and Evidence-Tier Analyses; Supplementary Figures |
| Validation design | Internal validation, external validation, prospective validation, real-time implementation, randomized validation, and quasi-experimental deployment evidence were distinguished. | Methods > Study Selection; Methods > Data Extraction; Results > Subgroup and Evidence-Tier Analyses |
| Performance metrics | AUROC was used as the primary quantitative synthesis metric because it was most consistently reported; sensitivity, specificity, accuracy, PPV, NPV, and other metrics were extracted where available. | Methods > Data Extraction; Methods > Statistical Analysis and Meta-Analysis; Table 1 |
| Calibration and threshold reporting | Calibration, threshold selection, and threshold-specific sensitivity/specificity were assessed where reported and identified as inconsistently reported across studies. | Methods > Risk-of-Bias and Applicability Assessment; Results > Risk-of-Bias and Applicability Assessment |
| Missing data and imputation | Missing-data handling and imputation strategies were extracted where reported and discussed as a major source of risk of bias and applicability concern. | Methods > Data Extraction; Results > Risk-of-Bias and Applicability Assessment; Discussion > Limitations |
| Explainability and transparency | Explainability, model transparency, availability of preprocessing pipelines, and code or data availability were considered in the risk-of-bias/applicability assessment. | Methods > Risk-of-Bias and Applicability Assessment; Table 2 |
| Deployment and workflow context | Prospective implementation and deployment studies, including NAVOY Sepsis, TREWS, COMPOSER, Sepsis ImmunoScore, and Epic Sepsis Model validation studies, were summarized narratively rather than pooled with retrospective studies. | Results > Subgroup and Evidence-Tier Analyses; Discussion > Comparison With Prior Work |
| Evidence level and clinical utility | The manuscript distinguishes retrospective discrimination performance from prospective workflow evaluation, randomized validation, and clinical effectiveness evidence. | Abstract; Results > Subgroup and Evidence-Tier Analyses |
| Bias, fairness, and generalizability | The manuscript discusses transportability, institutional variability, care-setting differences, alert burden, and potential bias from heterogeneous data sources. | Discussion; Limitations |
| Human oversight and clinical decision support | The manuscript avoids implying autonomous decision-making and frames AI models as potential decision-support tools requiring clinician review and prospective validation. | Discussion; Conclusions |
| Generative AI disclosure | The manuscript includes a separate statement disclosing use of generative AI tools for language editing and formatting support, if applicable. | Generative AI Disclosure |

**Abbreviations**: AI/ML, artificial intelligence/machine learning; AUROC, area under the receiver operating characteristic curve; DL, deep learning; ED, emergency department; ICU, intensive care unit; NPV, negative predictive value; PPV, positive predictive value. Prospective implementation and deployment studies, including NAVOY Sepsis [29], TREWS [33], COMPOSER [35], Sepsis ImmunoScore [36], and Epic Sepsis Model validation studies [37, 38], were summarized narratively rather than pooled with retrospective studies.

**Figure S5**. Exploratory Deeks’ funnel plot asymmetry test among studies with sufficient diagnostic-accuracy data

**Note**. Deeks’ funnel plot asymmetry test was performed among studies with sufficient threshold-specific diagnostic-accuracy data to estimate diagnostic odds ratios. The exploratory analysis included 9 studies, and the test did not indicate statistically significant funnel plot asymmetry or small-study effects (P=.167). Because many studies reported AUROC only, this analysis was interpreted as an available-data sensitivity analysis rather than a definitive publication-bias assessment.

**References**

1. Burdick H, Pino E, Gabel-Comeau D, et al. Validation of a machine learning algorithm for early severe sepsis prediction: a retrospective study predicting severe sepsis up to 48 h in advance using a diverse dataset from 461 US hospitals. BMC Med Inform Decis Mak. 2020;20:276. doi:10.1186/s12911-020-01284-x.
2. Barghi B, Azadeh-Fard N. Predicting risk of sepsis, comparison between machine learning methods: a case study of a Virginia hospital. Eur J Med Res. 2022;27:213. doi:10.1186/s40001-022-00843-4.
3. Mahyoub MA, Yadav RR, Dougherty K, et al. Development and validation of a machine learning model integrated with the clinical workflow for early detection of sepsis. Front Med (Lausanne). 2023;10:1284081. doi:10.3389/fmed.2023.1284081.
4. Kwon JM, Lee YR, Jung MS, et al. Deep-learning model for screening sepsis using electrocardiography. Scand J Trauma Resusc Emerg Med. 2021;29:145. doi:10.1186/s13049-021-00953-8.
5. Aşuroğlu T, Oğul H. A deep learning approach for sepsis monitoring via severity score estimation. Comput Methods Programs Biomed. 2021;198:105816. doi:10.1016/j.cmpb.2020.105816.
6. Gupta A, Liu T, Shepherd S. Clinical decision support system to assess the risk of sepsis using Tree Augmented Bayesian networks and electronic medical record data. Health Informatics J. 2020;26(2):841-861. doi:10.1177/1460458219852872.
7. Kok C, Jahmunah V, Oh SL, et al. Automated prediction of sepsis using temporal convolutional network. Comput Biol Med. 2020;127:103957. doi:10.1016/j.compbiomed.2020.103957.
8. Barton C, Chettipally U, Zhou Y, et al. Evaluation of a machine learning algorithm for up to 48-hour advance prediction of sepsis using six vital signs. Comput Biol Med. 2019;109:79-84. doi:10.1016/j.compbiomed.2019.04.027.
9. Al-Mualemi BY, Lu L. A deep learning-based sepsis estimation scheme. IEEE Access. 2021;9:5442-5452. doi:10.1109/ACCESS.2020.3043732.
10. Oei SP, van Sloun RJG, van der Ven M, et al. Towards early sepsis detection from measurements at the general ward through deep learning. Intell Based Med. 2021;5:100042. doi:10.1016/j.ibmed.2021.100042.
11. Bedoya AD, Futoma J, Clement ME, et al. Machine learning for early detection of sepsis: an internal and temporal validation study. JAMIA Open. 2020;3(2):252-260. doi:10.1093/jamiaopen/ooaa006.
12. Lauritsen SM, Kalør ME, Kongsgaard EL, et al. Early detection of sepsis utilizing deep learning on electronic health record event sequences. Artif Intell Med. 2020;104:101820. doi:10.1016/j.artmed.2020.101820.
13. Kam HJ, Kim HY. Learning representations for the early detection of sepsis with deep neural networks. Comput Biol Med. 2017;89:248-255. doi:10.1016/j.compbiomed.2017.08.015.
14. Ghias N, Haq SU, Arshad H, et al. Using machine learning algorithms to predict sepsis and its stages in ICU patients. Clinical Pediatrics and Mother Health. 2023;2(6). doi:10.31579/2835-2971/033.
15. Li J, Xi F, Yu W, et al. Real-time prediction of sepsis in critical trauma patients: machine learning-based modeling study. JMIR Form Res. 2023;7:e42452. doi:10.2196/42452.
16. Hu F, Zhu J, Zhang S, et al. A predictive model for the risk of sepsis within 30 days of admission in patients with traumatic brain injury in the intensive care unit: a retrospective analysis based on MIMIC-IV database. Eur J Med Res. 2023;28:290. doi:10.1186/s40001-023-01255-8.
17. Desautels T, Calvert J, Hoffman J, et al. Prediction of sepsis in the intensive care unit with minimal electronic health record data: a machine learning approach. JMIR Med Inform. 2016;4(3):e28. doi:10.2196/medinform.5909.
18. Zhang L, Huang T, Xu F, et al. Prediction of prognosis in elderly patients with sepsis based on machine learning (random survival forest). BMC Emerg Med. 2022;22:26. doi:10.1186/s12873-022-00582-z.
19. Scherpf M, Gräßer F, Malberg H, et al. Predicting sepsis with a recurrent neural network using the MIMIC III database. Comput Biol Med. 2019;113:103395. doi:10.1016/j.compbiomed.2019.103395.
20. Moor M, Bennett N, Plečko D, et al. Predicting sepsis using deep learning across international sites: a retrospective development and validation study. EClinicalMedicine. 2023;62:102124. doi:10.1016/j.eclinm.2023.102124.
21. Mollura M, Lehman LWH, Mark RG, et al. A novel artificial intelligence based intensive care unit monitoring system: using physiological waveforms to identify sepsis. Philos Trans A Math Phys Eng Sci. 2021;379(2212):20200252. doi:10.1098/rsta.2020.0252.
22. Wang Z, Yao B. Multi-branching temporal convolutional network for sepsis prediction. IEEE J Biomed Health Inform. 2022;26(2):876-887. doi:10.1109/JBHI.2021.3092835.
23. Duan Y, Huo J, Chen M, et al. Early prediction of sepsis using double fusion of deep features and handcrafted features. Appl Intell (Dordr). 2023;53:17903-17919. doi:10.1007/s10489-022-04425-z.
24. Delahanty RJ, Alvarez J, Flynn LM, et al. Development and evaluation of a machine learning model for the early identification of patients at risk for sepsis. Ann Emerg Med. 2019;73(4):334-344. doi:10.1016/j.annemergmed.2018.11.036.
25. Yuan KC, Tsai LW, Lee KH, et al. The development an artificial intelligence algorithm for early sepsis diagnosis in the intensive care unit. Int J Med Inform. 2020;141:104176. doi:10.1016/j.ijmedinf.2020.104176.
26. Kamaleswaran R, Sataphaty SK, Mas VR, et al. Artificial intelligence may predict early sepsis after liver transplantation. Front Physiol. 2021;12:692667. doi:10.3389/fphys.2021.692667.
27. Goh KH, Wang L, Yeow AYK, et al. Artificial intelligence in sepsis early prediction and diagnosis using unstructured data in healthcare. Nat Commun. 2021;12:711. doi:10.1038/s41467-021-20910-4.
28. Steinbach D, Ahrens PC, Schmidt M, et al. Applying machine learning to blood count data predicts sepsis with ICU admission. Clin Chem. 2024;70(3):506-515. doi:10.1093/clinchem/hvae001.
29. Persson I, Macura A, Becedas D, et al. Early prediction of sepsis in intensive care patients using the machine learning algorithm NAVOY Sepsis, a prospective randomized clinical validation study. J Crit Care. 2024;80:154400. doi:10.1016/j.jcrc.2023.154400.
30. Ivanov O, Molander K, Dunne R, et al. Detection of sepsis during emergency department triage using machine learning. arXiv. Preprint posted online April 15, 2022. arXiv:2204.07657. doi:10.48550/arXiv.2204.07657.
31. Taneja I, Damhorst GL, Lopez-Espina C, et al. Diagnostic and prognostic capabilities of a biomarker and EMR-based machine learning algorithm for sepsis. Clin Transl Sci. 2021;14(4):1578-1589. doi:10.1111/cts.13030.
32. Henry KE, Hager DN, Pronovost PJ, et al. A targeted real-time early warning score (TREWScore) for septic shock. Sci Transl Med. 2015;7(299):299ra122. doi:10.1126/scitranslmed.aab3719.
33. Henry KE, Adams R, Parent C, et al. Factors driving provider adoption of the TREWS machine learning-based early warning system and its effects on sepsis treatment timing. Nat Med. 2022;28(7):1447-1454. doi:10.1038/s41591-022-01895-z.
34. Yu SC, Gupta A, Betthauser KD, et al. Sepsis prediction for the general ward setting. Front Digit Health. 2022;4:848599. doi:10.3389/fdgth.2022.848599.
35. Boussina A, Shashikumar SP, Malhotra A, et al. Impact of a deep learning sepsis prediction model on quality of care and survival. npj Digit Med. 2024;7:14. doi:10.1038/s41746-023-00986-6.
36. Bhargava A, López-Espina C, Schmalz L, et al. FDA-authorized AI/ML tool for sepsis prediction: development and validation. NEJM AI. 2024;1(12):AIoa2400867. doi:10.1056/AIoa2400867.
37. Wong A, Otles E, Donnelly JP, et al. External validation of a widely implemented proprietary sepsis prediction model in hospitalized patients. JAMA Intern Med. 2021;181(8):1065-1070. doi:10.1001/jamainternmed.2021.2626.
38. Wong A, Currey D, Schwinne M, et al. Multicenter prospective validation of an updated proprietary sepsis prediction model. JAMA Netw Open. 2026;9(2):e260181. doi:10.1001/jamanetworkopen.2026.0181.
